# Does axial view still play an important role in dealing with calcaneal fractures?

**DOI:** 10.1186/s12893-015-0004-6

**Published:** 2015-03-08

**Authors:** Tao Zhang, Wei Chen, Yanling Su, Haili Wang, Yingze Zhang

**Affiliations:** Department of Orthopaedic Surgery, the Third Hospital of Hebei Medical University, NO.139 Ziqiang Road, Shijiazhuang, 050051 P R China; Key Laboratory of Orthopaedic Biomechanics of Hebei Province, The Third Hospital of Hebei Medical University, NO.139 Ziqiang Road, Shijiazhuang, 050051 P R China; Orthopaedic Research Institution of Hebei Province, Shijiazhuang, 050051 China

**Keywords:** Calcaneal fractures, Axial view, Widening of calcaneus

## Abstract

**Background:**

The study aimed to analyze the role of axial view in different phases of treatment and demonstrate whether axial view is still useful in evaluating the calcaneal fractures.

**Methods:**

156 patients with suspected unilateral calcaneal fractures were enrolled in the study, axial and lateral view of the affected foot and single axial view of the unaffected foot were gained. 16 were excluded due to unsatisfying axial radiograph. The remain 140 patients were eventually included into the study. Two separate assessments were conducted on two occasions with a three weeks interval to diagnose fractures. Lateral views were assessed firstly, and lateral combined with axial views were assessed three weeks later. Each of the 140 sets was evaluated by one of 6 surgeons randomly. Sensitivity and specificity value were compared between the two assessments. A new value Z which can directly reflect the degree of bulge on the calcaneal lateral wall on the axial view were introduced into the study. Z value of the 140 unaffected feet were measured. Data of another group of 31 patients who confirmed their lateral hindfoot pain caused by widening of calcaneus was reviewed. Liner regression was employed to analyze the relationship between angle Z and the severity of lateral pain.

**Results:**

According to the two assessments, without axial view, specificity value will be significantly lower in diagnosing calcaneal fractures (*p* = 0.024) and sensitivity value will be significantly lower in distinguishing intra-articular fractures (*p* < 0.001). The normal threshold of angle Z was estimated from 98.06° to 100.64° (p <0.001). Liner regression shows that the lateral hindfoot pain will obviously aggravate along with the increasing of angle Z value (p <0.001).

**Conclusions:**

Axial view is useful in diagnosing a patient with suspected calcaneal fracture especially for distinguishing intra-articular fractures and selection for CT scan. With the introduction of angle Z, axial view can get excellent performance in intra-operative assessment as well as in post-operative follow up procedure. Axial view can still play an irreplaceable role in assessing and evaluating calcaneal fractures, and can be employed as an essential reference during surgical procedure .

**Electronic supplementary material:**

The online version of this article (doi:10.1186/s12893-015-0004-6) contains supplementary material, which is available to authorized users.

## Background

Calcaneal fractures are the most commonly foot injury encountered by orthopedic surgeon, which comprise 1%-2% of all fractures [1-4]. Lateral and axial views are the standard plain radiographs in assessing calcaneal fractures in each phase of the treatment [5]. Lateral view has gained popularity in diagnosis of calcaneal injury [6]. Böhler angle [6] and Gissane angle can also be assessed with a lateral view, which are the two typical indexes used to diagnose and evaluate heel fracture [7-9]. For those who were suspected with an intra-articular calcaneal injury from lateral view, computed tomography (CT) scanning and three-dimension reconstruction can precisely demonstrate the existence of fracture and amount of intra-articular displacement. Usually, the axial view was used to evaluate widening and varus/valgus alignment of the hindfoot [5]. However, with the development of imaging technology, some believe axial view can be abandoned for its useless to diagnosis and treatment, since what can gain from axial view can also be gained from CT scans and even better [10]. Utukuri et al. [11] suggested in a research paper that axial view requires an additional radiation dosage and is difficult and painful to obtain, while not adding significant value to the evaluation and diagnosis of calcaneal fractures to be routinely requested. Does axial view still has an unique role in current treatment process of calcaneal fractures or just an out of fashion technique which should be abandoned? We conduct the study to test the value of axial view in diagnosing calcaneal fracture and in differentiating intra and extra-articular fracture by analyze the sensitivity and specificity. We also introduce a new value angle Z into the study to evaluate the bulging of calcaneus on axial. The other aim of the current study is identify the normal threshold of angle Z, and further demonstrate the relationship between Z angle and lateral hindfoot pain caused by calcaneal fractures.

## Methods

Retrospective review was performed on a, previously identified, cohort of 156 patients with suspected calcaneal fractures, all patients had a unilateral foot injury, were older than 18 years, without polytraumatic injuries of the ipsilateral lower limbs or severe medical ailments. All patients underwent calcaneal lateral and axial X-rays of the injured foot and single axial X-ray radiograph of the unaffected foot. Among the 156 patients, 16 were excluded due to unsatisfying axial radiograph before assessment. The remaining 140 patients, including 122 males and 18 females with a mean age 39.2 years (range, 19-61 years), were included for evaluation and analysis. At the first stage, the calcaneal lateral radiographs of the 140 sets were assessed by a group of 6 senior orthopedic surgeons. Six surgeons gain a number from 1 to 6, and each of the 140 sets were evaluated by one of the 6 randomly. During the examine process, a form with two questions should be filled out. Q1: Is there a calcaneal fracture? Q2: extra- or intra-articular fracture? In all patients, CT scan were subsequently obtained to confirm the diagnosis. At the second stage, the radiographs were rearranged three weeks later, and assessed by the same group of surgeons with both lateral and axial view.

**Figure 1:**
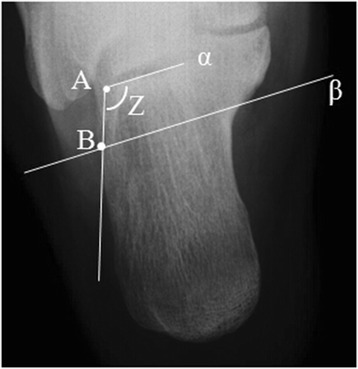
**The schematic drawing of the modality of measurement of Z angle.** Point A was the lateral end of the posterior facet of talus. A straight line α was made through point A and parallel to subtalar joint. Straight line β was parallel to subtalar joint and located at the level just below sustentaculum tali in the axial view. Point B was the intersection of line β and the lateral wall of calcaneus. Angle Z is formed by two intersecting lines: line α and line AB.

In this study, angle Z was served as a critical index to assess axial radiographs. As Figure 1 demonstrates, point A was the lateral end of the posterior facet of calcaneus. A straight line α was made through point A and parallel to subtalar joint. Line β was parallel to line α and located at the level just below sustentaculum tali in axial view. Point B was the intersection of line β and the lateral wall of calcaneus. Angle Z is formed by two intersecting lines: line α and line AB (Figure 1). Angle Z of the 140 sets axial views of the unaffected feet were identified, measured and recorded.

Another 31 patients who suffered from sustained isolate lateral hindfoot pain caused by malunion of calcaneal fractures after conservative treatment were included in the study. The source of pain was confirmed by injecting anesthetic into subtalar joint. Anesthetic technique with a minimal amount of local anesthetic was performed to the skin around injection point. The patients were then asked to perform activities which may elicit their pain, those who underwent immediate relief of pain after injection were excluded. Then patients were processed to anesthetic technique into peroneal tendon sheath to further identify the painful source. NPRS (Numerical Pain Rate Scale) was employed to evaluate the lateral hindfoot pain of the 31 patients, with other sources of pain were blocked by anesthetic. The severity of lateral pain slight pain (NPRS 0-3), moderate pain(NPRS 4-6) and severe pain (NPRS 7-10) were assigned a value of 1, 2 and 3 respectively and recorded for further analysis, Angle Z of the 31 patients were measured and recorded as well on axial view.

The study was approved by the Institutional Review Board of the Third Hospital of Hebei Medical University (Shijiazhuang, China). All patients included in the study signed the informed consents.

### Statistical analysis

The data was analyzed with SPSS 13.0 for Windows (SPSS Inc., Chicago, IL, USA). Categorical data was statistically analyzed by means of chi-square test or Fischer’s exact test (n < 40 or T < 1). Liner regression was employed to analyze the relationship between angle Z value and the values of Numerical Pain Rate Scale. Differences were regarded as statistically significant when *p* < 0.05.

## Result

According to CT scan, 97 of the 140 patients had a confirmed calcaneal fracture. Among the 97 patients with calcaneal fracture, 79 were intra-articular and the other 18 were either calcaneal tuberosity fractures or fractures involve calcaneocuboid joint. Table 1 shows the summarized datas of sensitivity and specificity values for the lateral view alone and lateral combine with axial views of each surgeon. Sensitivity and specificity value of the two groups were compared in Table 2. The sensitivity value and specificity value of single lateral view group are 94.85% and 79.07% respectively,which are 98.97% and 95.35% in combined lateral and axial group for diagnosing calcaneal fractures. For differentiating intra and extra-articular fractures, the two values are 69.62%, 94.44% in single group and 92.41%, 83.33% in combined group. It reveals that the sensitivity of single lateral view group is similar to the combined lateral and axial view group (*p* > 0.05) for diagnosing fractures but significantly lower in distinguishing intra/extra articular fractures (*p* < 0.001). In contrast, the specificity of the combined lateral and axial view group is significantly higher for diagnosing fractures (*p* = 0.024) but is similar to single lateral view group when distinguishing intra/extra articular fractures (*p* > 0.05).

**Table 1:** **Result of radiograph accessing**

		**M1**	**M2**	**M3**	**M4**	**M5**	**M6**	**Total**
For diagnosing calcaneal fracture								
Single lateral	Sensitivity	94.44	88.89	100	100	93.75	93.75	94.85%
	Specificity	83.33	85.71	75	66.67	71.43	100	79.07%
Lateral and axial	Sensitivity	100	100	100	100	100	94.44	98.97%
	Specificity	100	88.89	100	85.71	100	100	95.35%
For distinguishing intra/extra articular fracture								
Single lateral	Sensitivity	66.67	75	72.73	63.64	64.29	75	69.62%
	Specificity	100	100	80	100	100	100	94.44%
Lateral and axial	Sensitivity	92.86	100	85.71	100	84.62	92.31	92.41%
	Specificity	100	50	66.67	100	100	80	83.33%

**Table 2:** **Results of comparison**

**For diagnosing calcaneal fracture**				**For diagnosing intra-articular fracture**			
Sensitivity	Single lateral	94.85%	*P* > 0.05	Sensitivity	Single lateral	69.62%	*P < 0.001*
	Lateral and axial	98.97%			Lateral and axial	92.41%	
Specificity	Single lateral	79.07%	*P* = 0.024	Specificity	Single lateral	94.44%	*P* > 0.05
	Lateral and axial	95.35%			Lateral and axial	83.33%	

The average angle Z value of the 140 unaffected feet is 99.35 ± 7.73°range from 83.0°to 119.7°. The 95% confidence interval of the overall average value is estimated from 98.06° to 100.64° (*p* <0.001).

**Figure 2:**
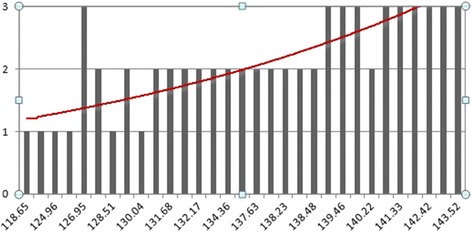
**Trend Chart for liner regression.** The severity of lateral pain will obviously aggravate along with the increasing of angle Z value.

According to the severity of lateral pain, there were 6 cases of slight pain, 15 cases of moderate pain and 10 cases of severe pain respectively. Liner regression analysis was tested to be statistically significant (F = 43.25, p < 0.001). The angle Z values were found to be associated with severity of lateral hindfoot pain (P < 0.001). The severity of lateral hindfoot pain has an obviously increasing tendency along with the adding of angle Z value (Figure 2).

## Discussion

Extensive use of CT scan extremely challenges the vital role of axial view in assessing calcaneal fractures [12,13]. CT scans combined with three-dimensional reconstruction are very helpful either in assessment of the fracture morphology or in planning the surgical protocol [14]. Lateral view of calcaneus is of proven value in diagnosing patients with suspected calcaneal fractures, and Böhler and Gissane angle can be also gained with it [7,15,16]. However, how can axial view be of any use to surgeon nowadays? Widening of the calcaneus can be evaluated via CT scans, and the severity of fracture and varus/valgus alignment of the hindfoot can be also assessed and even better by three-dimension reconstruction of the affected foot.

**Figure 3:**
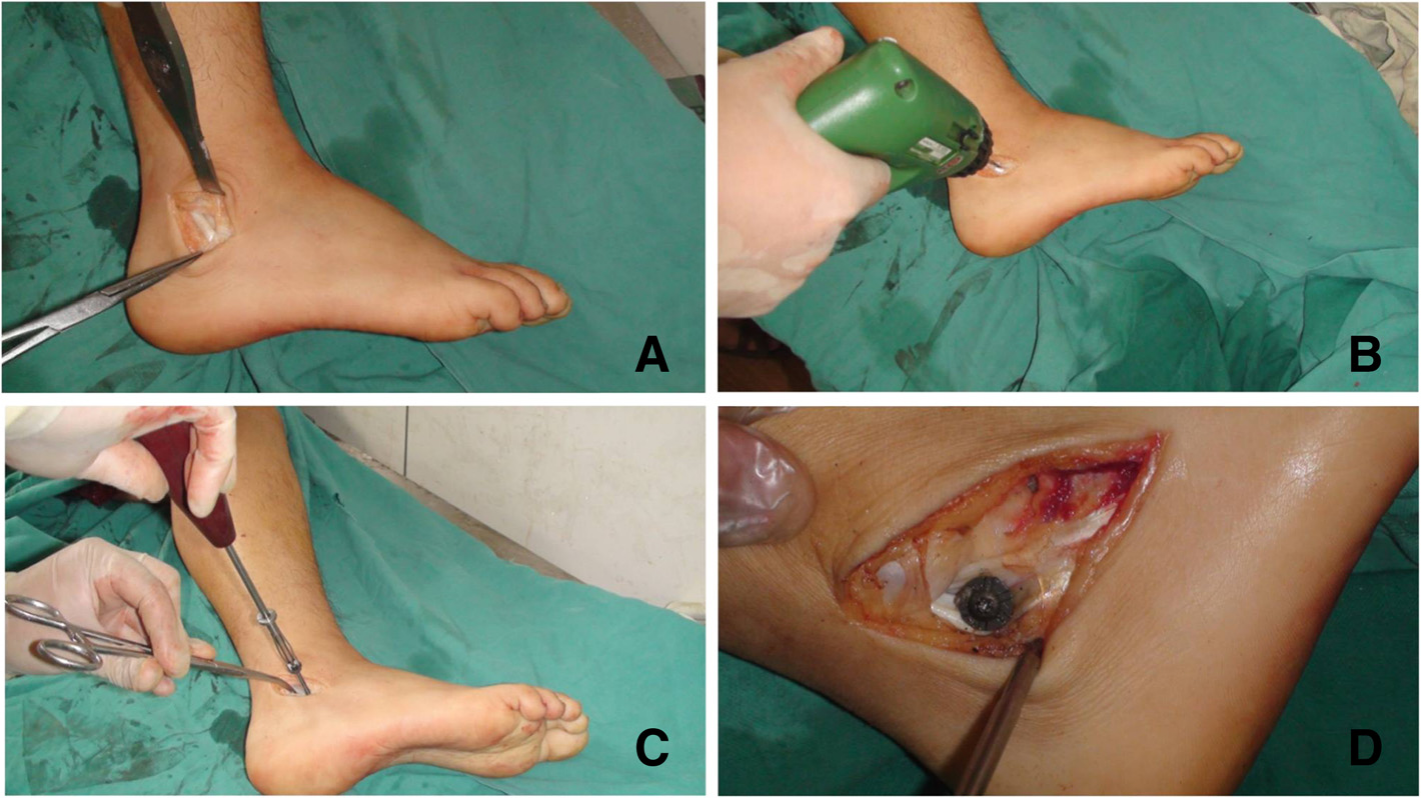
**Experiment on lower limb specimen. A)** The peroneal tendon sheath lay between retinaculum musculorum peroneorum superius and retinaculum musculorum peronaeorum inferius was exposed. **B)** A bicortical hole located below lateral malleolus and proximal to retinaculum musculorum peronaeorum inferius is made by the drill. The drill introduce through peroneal tendon sheath and perpendicular to the lateral wall. **C)** Screw was placed with a metal gasket. **D)** The gasket can cover majority of the tendon sheath below lateral malleolus.

**Figure 4:**
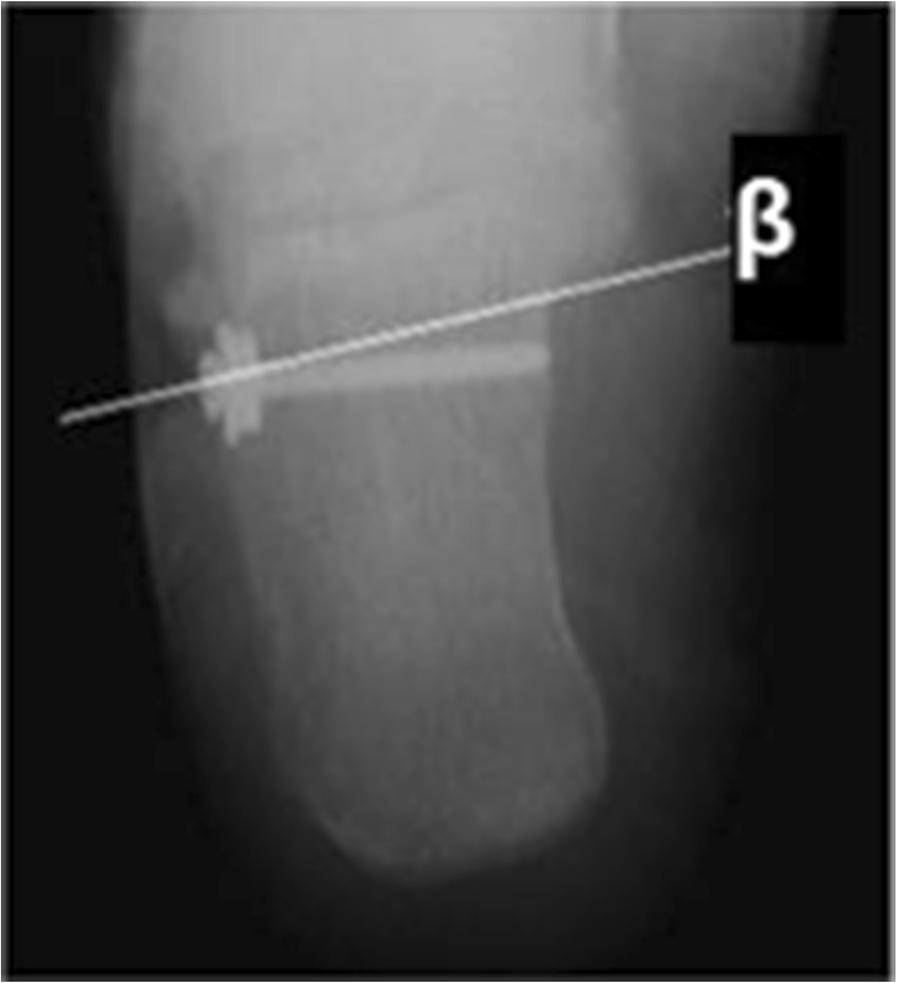
**Axial X-ray film after specimen experiment.** The axial view showed that central point of the gasket located at the level just below sustentaculum tali.

Can more information be gained from axial view? or it just supposed to be abandoned? We design the current study as an ongoing part of our former research [17] aim to figure out whether the axial view is still useful. As compression on the peroneal tendons caused by malunion of calcaneal fracture has been demonstrated to have a definite relationship with lateral pain, we introduced a measurable value - Angle Z in the current study to test the efficiency of axial view. The new value was born in a experiment on a limb specimen which was donated by an amputee suffered with malignant neoplasm of lower limb. The specimen was fixed on the operating table with the lateral malleolus facing up. An arc incision was made below lateral malleolus by a scalpel along the course of peroneal tendon sheath. Exposed the peroneal tendon sheath lay between retinaculum musculorum peroneorum superius and retinaculum musculorum peronaeorum inferius. Drill a bicortical hole located below lateral malleolus and proximal to retinaculum musculorum peronaeorum inferius. Drill should be introduced through peroneal tendon sheath and perpendicular to the lateral wall. Screw was placed with a metal gasket, and then the gasket can cover majority of the tendon sheath below lateral malleolus (Figure 3). An axial radiography of the experimental calcaneus was taken as the last step. Axial view showed that central point of the gasket just located on the parallel line (β) of subtalar joint below sustentaculum tali level (Figure 4). It revealed that bulging of the calcaneal lateral wall at line β level can directly reflect severity of irritation on peroneal tendon sheath. In addition, the sustentaculum tali suppose to be the most stable part of the calcaneus, since it is connected to the talus via strong medial and lateral talocalcaneal ligaments [18,19]. Therefore, we create the new value (angle Z) which can efficiently reflect degree of lateral wall bulging at line β level and try to demonstrate increasing of angle Z has a relationship to poor outcomes.

Below we analyze the clinical value of axial view in terms of three respective aspects, diagnosing process, intra-operative and post-operative process. At the very beginning of diagnosing suspected calcaneal fracture, plain radiograph can efficiently distinguish real fracture from others as a quick means of method. As it showned in Table 2, the specificity value of single lateral group is significantly lower for diagnosing fractures (*p* < 0.05) than combined lateral and axial view group, which means intact calcaneuses sometimes are difficult to rule out. The inefficiency of excluding intact calcaneus may lead to an increasing use of computed tomography scan which will not only increase unnecessary radiation dosage but also waste manpower and financial resources. In addition, the results also point out that sensitivity value of single lateral view group is significantly lower than combined lateral and axial view group (*p* < 0.05) for distinguishing intra/extra-articular fractures. It indicates that intra-articular fractures may be ignored if only lateral calcaneal view can be referenced [Additional files 1 and 2]. Overlooking an intra-articular fracture sometimes bring about conservative treatment to patients who should receive surgical procedure or proceed to CT scan. Utukuri et al [11] reported in a previous study that the axial view does not appear to add sufficiently to the diagnosis and evaluation of calcaneal fractures. The different results may due to increased sample size, random procedure and prolonged interval between two assessments in our study. Currently, CT scan is still an unconventional method in some regions and countries especially in developing country, and for selected patient, such as pregnant woman or child, application of CT scan are limited. Anyway, high radiation dosage of CT scan is indeed a potential risk for patients. All in all, Plain radiograph still play an irreparable role in diagnosing calcaneal fractures, and lateral combined with axial view will surely be more efficiency in diagnosing calcaneal fracture and distinguishing intra/extra articular fractures than single lateral view. Axial view should be performed combined with lateral view and radiograph of ankle for all suspected calcaneal fractures after ankle or hindfoot trauma to avoid missing diagnosis.

During operative process, the axial view used to be widely accepted as an essential method to evaluate the reduction quality of calcaneal fractures [20]. The width of calcaneus can be visually assessed with an axial view, and restore the width of calcaneus is the key point of prevent long-term lateral pain of the heel [21-23]. However, there has been a controversy for a long time, widening of which part can directly indicated a high risk of long-term lateral hindfoot pain [24,25]? Neither definite method of assessment nor measurable index which leads to diagnostic value of axial views decreased. As we mentioned above, a measurable value (angle Z) was introduced into our current study which can efficiently reflect degree of lateral wall bulging around the course of peroneal tendons below lateral malleolus. Result of liner regression analysis shows the severity of lateral pain will obviously aggravate along with the increasing of angle Z value (Figure 2). Additionally, lateral hindfoot pain correlated directly to peroneal tendon sheath impingement [17]. Therefore, Z value has a positive relationship with the severity of compression on the peroneal tendons after calcaneal fractures. In other words, angle Z value can be used not only to assess restore of width but also to evaluate the therapeutic effect as a measurable index. Angle Z value is easy to gain and should be recovered to the normal level (98.06° to 100.64°) during surgery process in order to improve long-term outcomes. In the mean time, varus / valgus alignment of the hindfoot can be observed on axial views. X-ray plain film is obviously easy to gain than intra-operative CT scan during operation even if in highly specialized and fully equipped trauma centre. Extra radiation dosage can also be avoided if patients can be treated with plain radiographs rather than proceed to CT examination [11], furthermore, operation time and infection risk should also be decreased. Although if available, a CT scan and 3D reconstruction is still the most precise way to evaluate calcaneal fractures.

During post-operative inpatient and outpatient follow up process, axial view can be employed to assess the loss of reduction combined with lateral calcaneus view. Böhler angle can be gained through lateral view and angle Z can be gained from axial view respectively. Synthetically analyze the two indexes (Böhler and Z angle) can predict the long-term outcomes more effectually than merely lateral view [26].

There are still some limitations in the current study. First, standard axial views are painful to obtain especially in diagnosing and post-operative procedure. Therefore, some datas in our study excluded due to a satisfying preoperative axial view can not be obtained. More standardized, convenient and painless procedure for axial view should be introduced in future studies to minimize the bias among different axial views. In addition, Numerical Pain Rate Scale (NPRS) which is an subjective evaluate system may compromise the reliability of results, even though we divided the NPRS values into three levels (slight, moderate and severe) to minimize the variability. Peroneal tenography suppose to be more objective in reflecting the severity of compression on peroneal tendon sheath than NPRS scale, however, it is hard to persuade all the patients to accept the invasive technique. Furthermore, more exquisitely designed prospective studies are needed to further confirm the relationship between ankle Z and long-term lateral pain after calcaneal fractures.

## Conclusion

Axial view is useful in diagnosing a patient with suspected calcaneal fracture especially for distinguishing intra-articular fractures and selection for CT scan. With the introduction of angle Z, axial view can get excellent performance in both intra-operative assessment and post-operative follow-up evaluation. Axial view can still play an irreplaceable role in assessing and evaluating calcaneal fractures, and can be employed as an essential reference during surgical procedure.

## Additional files

A pair of axial and lateral view of calcaneal fracture were provided to show the fracture type which is hard to be found on single lateral view. It is hard to be found on the lateral view if a fracture line lies on sagittal plane and without obvious displacement [see Additional file 1]. In contrast, axial view can show the sagittal fracture line clearly [see Additional file 2]. Additional file 1:
**Lateral view of the calcaneal fracture.**
Additional file 2:
**Axial view of the calcaneal fracture.**

## References

[1] Veltman ES, Doornberg JN, Stufkens SA (2013). Long-term outcomes of 1,730 calcaneal fractures: systematic review of the literature. J Foot Ankle Surg.

[2] Firoozabadi R, Kramer PA, Benirschke SK (2013). Plantar medial wounds associated with calcaneal fractures. Foot Ankle Int.

[3] Mitchell MJ, McKinley JC, Robinson CM (2009). The epidemiology of calcaneal fractures. Foot (Edinb).

[4] Matherne TH, Tivorsak T, Monu JU (2007). Calcaneal fractures: what the surgeon needs to know. Curr Probl Diagn Radiol.

[5] Rammelt S, Zwipp H (2004). Calcaneus fractures: facts, controversies and recent developments. Injury.

[6] Su Y, Chen W, Wu Z (2013). Böhler angle’s role in assessing the injury severity and functional outcome of internal fixation for displaced intra-articular calcaneal fractures:a retrospective study. BMC Surg.

[7] Knight JR, Gross EA, Bradley GH (2006). Boehler’s angle and the critical angle of Gissane are of limited use in diagnosing calcaneus fractures in the ED. Am J Emerg Med.

[8] Rammelt S, Barthel S, Biewener A (2003). Calcaneus fractures. Open reduction and internal fixation. Zentralbl Chir.

[9] Maskill JD, Bohay DR, Anderson JG (2005). Calcaneus fractures: a review article. Foot Ankle Clin.

[10] Sanders R (2000). Displaced intra-articular fractures of the calcaneus. J Bone Joint Surg Am.

[11] Utukuri MM, Knowles D, Smith KL (2000). The value of the axial view in assessing calcaneal fractures. Injury.

[12] Crosby LA, Fitzgibbons T (1990). Computerized tomography scanning of acute intra-articular fractures of the calcaneus. A new classification system. J Bone Joint Surg Am.

[13] Sanders R, Fortin P, DiPasquale T (1993). Operative treatment in 120 displaced intraarticular calcaneal fractures. Results using a prognostic computed tomography scan classification. Clin Orthop Relat Res.

[14] Ebraheim NA, Biyani A, Padanilam T (1996). A pitfall of coronal computed tomographic imaging in evaluation of calcaneal fractures. Foot Ankle Int.

[15] Eastwood DM, Gregg PJ, Atkins RM (1993). Intra-articular fractures of the calcaneum. Part I: Pathological anatomy and classification. J Bone Joint Surg (Br).

[16] Eastwood DM, Langkamer VG, Atkins RM (1993). Intra-articular fractures of the calcaneum. Part II: Open reduction and internal fixation by the extended lateral transcalcaneal approach. J Bone Joint Surg (Br).

[17] Chen W, Li X, Su Y (2011). Peroneal tenography to evaluate lateral hindfoot pain after calcaneal fracture. Foot Ankle Int.

[18] Della Rocca GJ, Nork SE, Barei DP (2009). Fractures of the sustentaculum tali: injury characteristics and surgical technique for reduction. Foot Ankle Int.

[19] Mahato NK (2011). Morphology of sustentaculum tali: Biomechanical importance and correlation with angular dimensions of the talus. Foot (Edinb).

[20] Wu Z, Su Y, Chen W (2012). Functional outcome of displaced intra-articular calcaneal fractures: a comparison between open reduction/internal fixation and a minimally invasive approach featured an anatomical plate and compression bolts. J Trauma Acute Care Surg.

[21] Braly WG, Bishop JO, Tullos HS (1985). Lateral decompression for malunited os calcis fractures. Foot Ankle.

[22] Miller WE (1983). Pain and impairment considerations following treatment of disruptive os calcis fractures. Clin Orthop Relat Res.

[23] Myerson M, Quill GE (1993). Late complications of fractures of the calcaneus. J Bone Joint Surg Am.

[24] Paley D, Hall H (1993). Intra-articular fractures of the calcaneus. A critical analysis of results and prognostic factors. J Bone Joint Surg Am.

[25] Schildhauer TA, Sangeorzan BJ (2002). Push screw for indirect reduction of severe joint depression-type calcaneal fractures. J Orthop Trauma.

[26] Loucks C, Buckley R (1999). Bohler’s angle: correlation with outcome in displaced intra-articular calcaneal fractures. J Orthop Trauma.

