# Supplementary figures and images for: Does axial view still play an important role in dealing with calcaneal fractures?
Source: BMC Surg. 2015 Mar 8;15:19. doi: 10.1186/s12893-015-0004-6 (PMC4359527; doi:10.1186/s12893-015-0004-6)

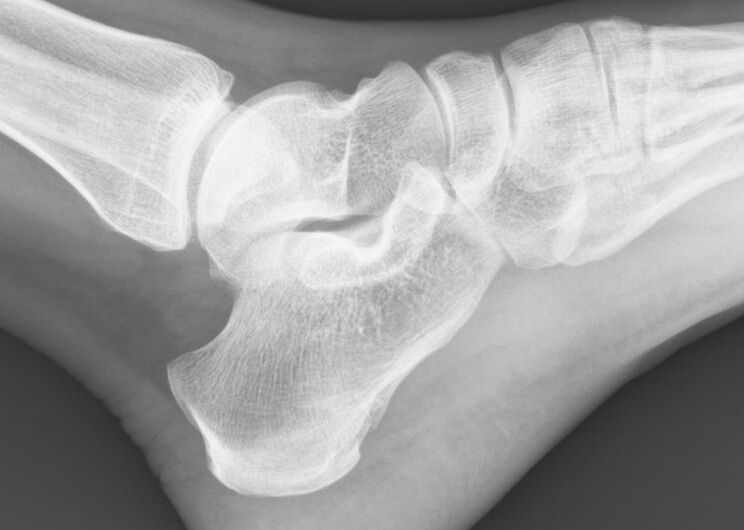

Supplement: Additional file 1: — Lateral view of the calcaneal fracture. [file 12893_2015_4_MOESM1_ESM.jpeg]

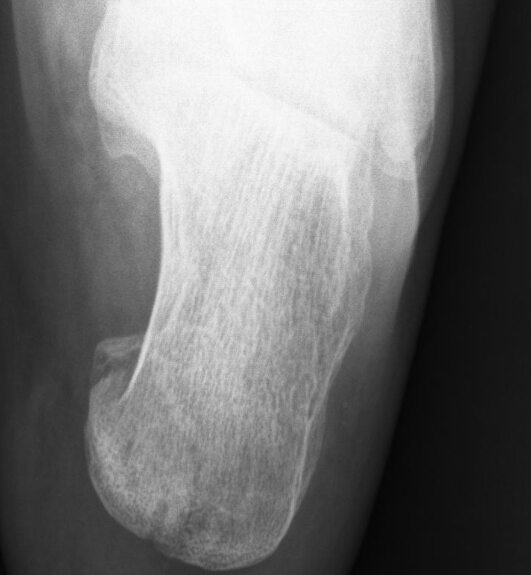

Supplement: Additional file 2: — Axial view of the calcaneal fracture. [file 12893_2015_4_MOESM2_ESM.jpeg]
